# Aqueous Level of ANGPTL4 Correlates with the OCTA Metrics of Diabetic Macular Edema in NPDR

**DOI:** 10.1155/2022/8435603

**Published:** 2022-01-19

**Authors:** Qing Xu, Chaoju Gong, Lei Qiao, Ruifang Feng, Haiyang Liu, Yalu Liu, Sujuan Ji, Yipeng Zhang, Shuang Wu, Suyan Li

**Affiliations:** Department of Ophthalmology, The Affiliated Xuzhou Municipal Hospital of Xuzhou Medical University, Xuzhou First People's Hospital, Xuzhou Eye Disease Prevention and Treatment Institute, Xuzhou, 221116 Jiangsu Province, China

## Abstract

**Purpose:**

To investigate the aqueous levels of angiogenic factors in nonproliferative diabetic retinopathy (NPDR) patients with diabetic macular edema (DME) and to ascertain their association with optical coherence tomography angiography (OCTA) metrics.

**Methods:**

This study enrolled 21 NPDR eyes with DME (NPDR/DME+), 17 NPDR eyes without DME (NPDR/DME-), and 16 diabetic eyes without retinopathy (DWR). Luminex bead-based multiplex array was used to measure the levels of 25 cytokines. OCTA system with a scan area of 3 × 3 mm was used to measure retinal thickness (RT), retinal volume (RV), superficial vessel density (SVD), deep vessel density (DVD), foveal avascular zone (FAZ) area, perimeter and acircularity index.

**Results:**

The levels of ANGPTL4 were significantly different among the three groups (*P* < 0.05), in which NPDR/DME+ group had the highest level and NPDR/DME- group had a higher level than the DWR group (all, *P* < 0.0167). OCTA examination showed that, compared with DWR and NPDR/DME- group, RT and RV increased and the whole/parafoveal DVD decreased in NPDR/DME+ group (all, *P* < 0.05). Meanwhile, NPDR/DME- group had lower parafoveal DVD than the DWR group (*P* < 0.05). Correlation analysis showed that the levels of ANGPTL4 were positively correlated with foveal/parafoveal RT and RV and negatively correlated with whole/parafoveal DVD in NPDR patients (all, *P* < 0.05). As the influencing factor of RT, RV, and DVD, every additional 10^3^ pg/ml of ANGPTL4 was associated with an increase in foveal and parafoveal RT of 4.299 *μ*m and 3.598 *μ*m, respectively. Every additional 10^6^ pg/ml of ANGPTL4 was associated with an increase in foveal and parafoveal RV of 3.371 mm^3^ and 17.705 mm^3^, respectively. Every additional 10^4^ pg/ml of ANGPTL4 was associated with a decrease in whole and parafoveal DVD of 1.705% and 1.799%, respectively.

**Conclusions:**

The level of ANGPTL4 in aqueous humor of NPDR patients with DME was significantly increased and ANGPTL4 might predict RT, RV, and parafoveal DVD of DME in NPDR patients.

## 1. Introduction

Diabetic retinopathy (DR) is one of the most destructive microvascular complications of diabetes mellitus. Intraocular neovascularization and diabetic macular edema (DME) are two major clinicopathologic features during the development and progression of DR [1, 2]. The occurrence of neovascularization is closely related to the duration of diabetes mellitus. Patients with recently diagnosed diabetes mellitus have a lower risk of proliferative diabetic retinopathy (PDR) involving neovascularization than those with longer duration [3]. DME is a major cause of visual impairment in DR patients, which can occur at any stage of DR, even at early and mild nonproliferative diabetic retinopathy (NPDR) stage [4]. Therefore, the prevention and early diagnosis of DME in NPDR are extremely important.

The pathogenesis of DME is multifactorial and remains unknown. Recent studies suggest that DME occurrence is induced by the breakdown of the blood-retina barrier (BRB) and the consequent increases in vascular permeability, vascular leakage, and fluid accumulation within the macula, which causes retina thickening, macular malfunction, and visual impairment [5]. Angiogenesis and inflammation play a critical role in the pathogenesis of DME involving many exudative cytokines [6–9].

As a potent angiogenesis factor, vascular endothelial growth factor (VEGF) can increase vascular permeability in DME pathogenesis [10]. At present, anti-VEGF therapy is a first-line treatment for DME. Studies have shown that VEGF inhibition effectively improved visual acuity and reduced macular thickness [11]. Nevertheless, the responses to anti-VEGF therapy were distinct in different cases. Specially, persistent DME did not improve even after several administrations of anti-VEGF drugs [4, 11]. The above findings reveal that other mechanisms, independent of VEGF, may also contribute to DME.

However, previous studies mainly focused on inflammatory factors in DME, but not angiogenic factors. Furthermore, the conclusions from these studies were only based on the comparison of cytokine levels between DME and nondiabetic control [7, 11, 12], which could not rule out the interference of diabetes and DR severities on cytokine level.

Here, we choose 25 angiogenic factors based on established and hypothesized angiogenesis pathway in DR and DME [13, 14], compared cytokines levels in the aqueous humor of NPDR patients with or without DME, and then investigated the effects of differentially expressed cytokines on optical coherence tomography angiography (OCTA) metrics to explore the potential molecular markers for DME in NPDR patients.

## 2. Methods

### 2.1. Study Subjects

This study included 21 eyes of 21 NPDR patients with DME (NPDR/DME+) who received intravitreal injection of anti-VEGF agents in the ophthalmology department of Xuzhou First People's Hospital from July 2017 to December 2018. 33 eyes of 33 senile cataract patients with diabetes mellitus who underwent phacoemulsification at the same time were enrolled, in which 17 eyes of 17 NPDR patients without DME (NPDR/DME-) and 16 eyes of 16 diabetic patients without retinopathy (DWR) were identified by slit-lamp biomicroscopy, fundus photography, and OCTA three days after the operation. DWR group served as controls. The inclusion criteria were as follows: (1) NPDR patients with DME, DME was defined with one or more of the follows: retinal thickening at or within 500 *μ*m of the macular center; hard exudates at or within 500 *μ*m of the macular center, also associated with adjacent retinal thickening; one or more zones of retinal thickening with one optic disc size, at least part of which within the range of one optic disc diameter in the macular center [15]. The diagnosis and classification of NPDR were based on the standards published by the international ophthalmological association [16]. (2) Senile cataract patients with type 2 diabetes mellitus who received phacoemulsification were diagnosed as NPDR without DME or DWR. Exclusion criteria were as follows: (1) proliferative diabetic retinopathy; (2) a history of vitreous hemorrhage, retinal detachment, intraocular surgery (except cataract surgery) or ocular trauma; (3) anti-VEGF or laser therapy previously; (4) complication with uveitis, glaucoma, optic nerve disease, or other eye diseases; (5) low signal strength index (SSI < 50), blink artifacts or motion. This study followed the Declaration of Helsinki and was approved by the Ethics Committee of Xuzhou First People's Hospital (approval number: xyyll [2017] 008). Informed consent was obtained from all patients.

### 2.2. Ophthalmic Examination

All patients underwent comprehensive ophthalmic examination, including visual acuity, intraocular pressure, slit-lamp biomicroscopy, fundus photography, and OCTA. The images were diagnosed by two independent doctors, and cases with a discrepancy were reviewed by the third doctor with a higher qualification. OCTA (Optovue, Inc., Fremont, CA, USA) was performed using the angio retina mode. For each eye, a 3 × 3 mm area centered on the fovea was scanned. Retinal thickness (RT) and retinal volume (RV) in the foveal and parafoveal area were automatically calculated by the built-in software from internal limiting membrane (ILM) to retinal pigment epithelium (RPE) layer. The fovea was defined as the circle area within the central 1 mm of the macula. Parafovea was defined as an area from the central 1 mm to the central 3 mm ring of the macular [17]. The OCTA images were automatically segmented to superficial capillary plexuses (SCP) and deep capillary plexuses (DCP) using the built-in software segmentation algorithm. The SCP was segmented with an inner boundary at 3 *μ*m beneath the ILM and an outer boundary at 15 *μ*m beneath the inner plexiform layer (IPL). The DCP was segmented with an inner boundary 15 *μ*m beneath the IPL and an outer boundary at 70 *μ*m beneath the IPL [18]. The vessel density values for the SCP and DCP in the whole, foveal, and parafoveal zones were calculated by the Angiovue Analytics built-in software. Vessel density was calculated as the percentage of pixels with flow signal above the preset decorrelation threshold in the defined region. FAZ area, perimeter, acircularity index, and FD-300 vessel density were automatically obtained via the FAZ assessment tool. FAZ surrounded by a continuous vascular closed ring was taken from ILM to outer plexiform layer (OPL). FD-300 was defined as a 300 *μ*m ring around the FAZ [17].

### 2.3. Sample Collection

Aqueous humor was collected before cataract surgery or intravitreal injection of anti-VEGF agents. After topical anesthesia, 100 *μ*L undiluted aqueous humor was withdrawn aseptically using an insulin syringe with a 30G needle at 1 mm inside the corneal limbus, which was placed in a 0.5 mL sterile Eppendorf tube and then stored at -80°C until measurement.

### 2.4. Measurement of Cytokines

Twenty-five cytokines, including epidermal growth factor (EGF), hepatocyte growth factor (HGF), heparin-binding EGF-like growth factor (HB-EGF), fibroblast growth factor 1 (FGF-1), FGF-2, FGF-19, FGF-21, FGF-23, granulocyte colony-stimulating factor (G-CSF), bone morphogenetic protein 9 (BMP-9), Endoglin, Endothelin-1, Leptin, Follistatin, *α*-Fetoprotein, FABP1, interleukin-8 (IL-8), Angiopoietin-2 (ANG-2), angiopoietin-like 3 (ANGPTL3), ANGPTL4, ANGPTL6, placental growth factor (PLGF), VEGF-A, VEGF-C, and VEGF-D (angiogenesis/growth factor panels, HAGP1MAG-12K and HLPPMAG-57K; Millipore Corporation, Billerica, MA, USA) were measured with Luminex bead-based multiplex array. All assays were performed strictly according to the manufacturer's guidelines, the detailed procedures of which were described in a previous study [19]. In brief, the assay buffer (25 *μ*L) was added to the background, standard, control, and sample wells. Each standard or control (25 *μ*L) was added into the appropriate wells. Aqueous humor (25 *μ*L) was added into the appropriate wells. Mixed beads (25 *μ*L) were added to each well. Seal, wrap with foil, and incubate with agitation on a plate shaker overnight at 4°C. Gently remove fluid and wash plate 3 times. Next, detection antibody (25 *μ*L) was added, and the plate was incubated for 1 hour at room temperature. Then, streptavidin-Phycoerythrin solution (25 *μ*L) was added to each well, and the plate was incubated for 30 minutes at room temperature. Gently remove well contents and wash plate 3 times. Sheath fluid (100 *μ*L) was added to all wells, and the beads were resuspended on a plate shaker for 5 minutes. Next, the plate was run on the FlexMAP 3D (Luminex) platform with xPONENT software. Median Fluorescent Intensity (MFI) was collected using Milliplex analyst 5.1 software. Standard curve for each cytokine was generated using the standards provided in the kits, and cytokine concentrations were obtained from the standard curves using a 5-parameter curve-fitting algorithm to transform the MFI values into concentrations.

### 2.5. Statistical Analyses

Statistical analysis was performed with SPSS 19.0. Shapiro-Wilk test was used to assess the normality of measurement variables. Normally distributed variables were expressed as mean ± standard deviation, whereas skewed distributed variables were expressed as median (Q1, Q3). Categorical variables were summarized as counts and percentage. Comparisons of categorical variables were performed using chi-squared test. One-way analysis of variance with post hoc least significant difference (LSD) multiple comparison tests was performed for normally distributed variables among the three groups. Kruskal-Wallis *H* test was performed for skewed variables among the three groups. Mann–Whitney *U* test was performed for skewed variables between two groups, and a *P* < 0.0167 (0.05/3) was considered significant for multiple comparisons. Spearman's rank correlation test was performed to assess the association between cytokine levels and the OCTA metrics. The correlation coefficient was tested by Student's *t* test and the cytokines with *P* < 0.05 were included for single-factor linear regression analysis, and the cytokines with *P* < 0.05 in the single factor linear regression were further included in the multiple linear regression model. RT (foveal and parafoveal), RV (foveal and parafoveal), and DVD (whole and parafoveal) were used as dependent variables, respectively, ANGPTL4 and VEGF-A were used as independent variables, stepwise multiple linear regression was used to evaluate the cytokines that affect OCTA metrics, and *P* < 0.05 was considered statistically significant.

## 3. Results

### 3.1. Demographic Characteristics

There were no significant differences in age, gender composition, body mass index (BMI), mean arterial pressure (MAP), fasting plasma glucose, HbAlc, and the duration of diabetes among the three groups (all, *P* > 0.05). There were no significant differences in severity degree of NPDR between NPDR/DME+ and NPDR/DME- groups (*P* > 0.05) (Table 1).

### 3.2. Comparison of Cytokine Levels in Aqueous Humor

The levels of ANGPTL4 among the three groups were significantly different from each other (*P* < 0.05). NPDR/DME+ group had the highest level of ANGPTL4 in the three groups, and NPDR/DME- group had a higher ANGPTL4 level than the DWR group (all, *P* < 0.0167). For HGF, IL-8, PLGF, and VEGF-A, NPDR/DME+ group had the highest levels in the three groups (all, *P* < 0.0167), but no significant difference between NPDR/DME- and DWR group was found (all, *P* > 0.0167). In addition, NPDR/DME+ group had a higher level of FGF-19 than the NPDR/DME- group (*P* < 0.0167), but no significant changes were detected in NPDR/DME+ and NPDR/DME- groups, compared with the DWR group (all, *P* > 0.0167). There were no significant differences of HB-EGF, FGF-2, Endothelin-1, Leptin, ANG-2, and VEGF-C among the three groups (all, *P* > 0.05), and the levels of EGF, FGF-1, FGF-21, FGF-23, G-CSF, BMP-9, Endoglin, Follistatin, AFP, FABP1, ANGPTL3, ANGPTL6, and VEGF-D were lower than the minimum detectable levels of the panel (Table 2).

### 3.3. Comparison of OCTA Metrics

**Figure 1:**
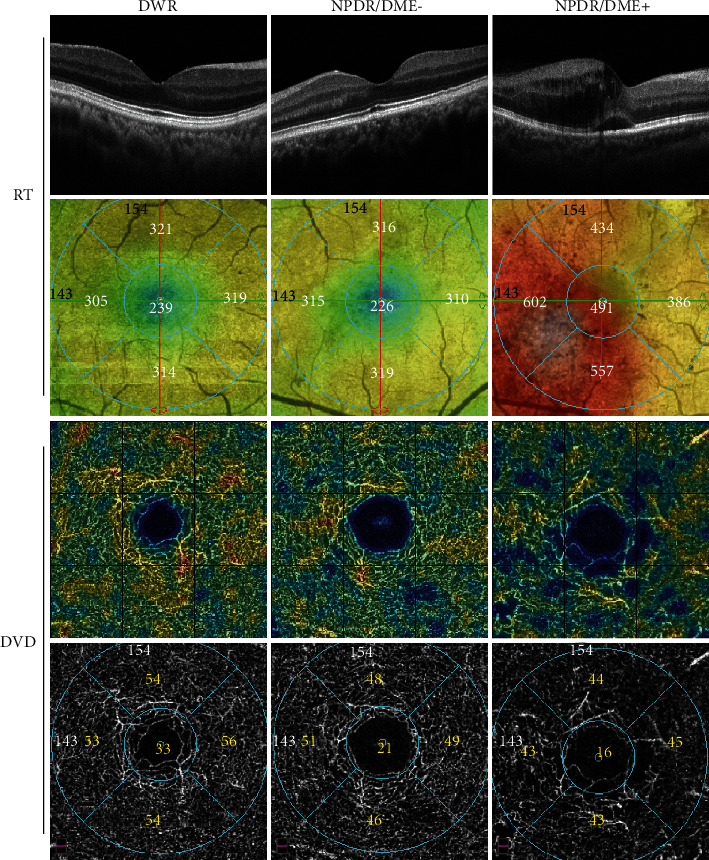
OCTA images of the three groups. Compared with DWR and NPDR/DME- groups, RT was significantly increased and DVD was significantly decreased in NPDR/DME+ groups.

Compared with DWR and NPDR/DME- group, RT and RV in NPDR/DME+ group were significantly increased, and the whole/parafoveal deep vessel densities (DVD) were reduced (Figure 1, Table 3). Besides, NPDR/DME- group had a lower parafoveal DVD than the DWR group (all, *P* < 0.05) (Table 3). There were no significant differences of superficial vessel density (SVD), foveal DVD, FD-300 vessel density, FAZ area, FAZ perimeter, and acircularity index among the three groups (all, *P* > 0.05) (Table 3).

### 3.4. Correlation between Cytokine Levels in Aqueous Humor and OCTA Metrics

For all the NPDR patients, correlations between the above differentially expressed cytokines including HGF, FGF19, IL-8, ANGPTL4, PLGF, VEGF-A, and the OCTA metrics including RT, RV, and DVD were analyzed. The levels of ANGPTL4 and VEGF-A were positively correlated with foveal RT (*rs* = 0.569, *P* < 0.001; *rs* = 0.528, *P* = 0.001), parafoveal RT (*rs* = 0.555, *P* < 0.001; *rs* = 0.437, *P* = 0.006), foveal RV (*rs* = 0.566, *P* < 0.001; *rs* = 0.530, *P* = 0.001), parafoveal RV (*rs* = 0.519, *P* = 0.001; *rs* = 0.389, *P* = 0.016), and negatively correlated with whole DVD (*rs* = −0.352, *P* = 0.030; *rs* = −0.373, *P* = 0.021) and parafoveal DVD (*rs* = −0.421, *P* = 0.008; *rs* = −0.422, *P* = 0.008). The level of PLGF was positively correlated with foveal RT (*rs* = 0.363, *P* = 0.025), parafoveal RT (*rs* = 0.352, *P* = 0.030), foveal RV (*rs* = 0.365, *P* = 0.024), and parafoveal RV (*rs* = 0.326, *P* = 0.046) but not with DVD (*P* > 0.05). The level of FGF-19 was positively correlated with parafoveal RT (*rs* = 0.332, *P* = 0.042) but not with foveal RT, RV, and DVD (all, *P* > 0.05). There were no correlations between HGF, IL-8, and RT, RV, and DVD, respectively (all, *P* > 0.05) (Table 4).

### 3.5. The Effect of Cytokines on OCTA Metrics

VEGF-A and ANGPTL4 with *P* < 0.05 in the single factor linear regression were further included in the multiple linear regression model (Table S1). Multiple regression analysis showed that the level of ANGPTL4 was an influencing factor for RT, RV, and DVD. The regression equations were fitted as follows: foveal RT = 223.422 + 4.299 × 10^−3^ × ANGPTL4, parafoveal RT = 294.302 + 3.598 × 10^−3^ × ANGPTL4, foveal RV = 0.176 + 3.371 × 10^−6^ × ANGPTL4, parafoveal RV = 1.838 + 17.705 × 10^−6^ × ANGPTL4, whole DVD = 46.587 − 1.705 × 10^−4^ × ANGPTL4, and parafoveal DVD = 49.265 − 1.799 × 10^−4^ × ANGPTL4. Every additional 10^3^ pg/ml of ANGPTL4 was associated with an increase in foveal and parafoveal RT of 4.299 *μ*m and 3.598 *μ*m, respectively. Every additional 10^6^ pg/ml of ANGPTL4 was associated with an increase in foveal and parafoveal RV of 3.371 mm^3^ and 17.705 mm^3^, respectively. Every additional 10^4^ pg/ml of ANGPTL4 was associated with a decrease in whole and parafoveal DVD of 1.705% and 1.799%, respectively. The level of VEGF-A had no effect on RT, RV, and DVD (all, *P* ≥ 0.05) (Table 5).

## 4. Discussion

Our study found that the levels of HGF, FGF-19, IL-8, ANGPTL4, PLGF, and VEGF-A increased in the aqueous humor of NPDR/DME+ patients, compared with NPDR/DME- and DWR patients. Notably, the levels of VEGF-A and ANGPTL4 were correlated with RT, RV, and DVD, in which single factor linear regression showed that both VEGF-A and ANGPTL4 were the influencing factors for RT, RV, and DVD. These findings supported the previous reports that VEGF-A contributed to the pathogenesis of DME [20, 21]. Cytokines with *P* < 0.05 in the single factor regression were further included in the multiple linear regression model. Although the *P* value for VEGF-A was 0.05, we still considered VEGF-A as an influencing factor for macular edema in NPDR patients. Moreover, in the models with multiple cytokines, VEGF-A had a greater impact on foveal RT and RV than other OCTA metrics.

Our study also found that the three groups were different from each other in the levels of ANGPTL4. Kwon et al. reported that both NPDR and PDR groups with similar severities of DME had higher levels of ANGPTL4 than the cataract controls, and the PDR group had a higher level than the NPDR group [22]. Here, we found that both DME+ and DME- groups with similar severities of NPDR had higher ANGPTL4 levels than the DWR group, and the DME+ group had a higher level than the DME- group. These results suggested that ANGPTL4 was also associated with of DME. Multiple regression analysis revealed that the level of ANGPTL4 in aqueous humor was an influencing factor for RT, RV, and DVD. NPDR patients with high levels of ANGPTL4 in the aqueous humor had higher foveal/parafoveal RT and RV and lower whole/parafoveal DVD than the patients with low levels of ANGPTL4.

As an angiogenesis factor, ANGPTL4 promotes the pathological processes of diverse eye diseases by enhancing angiogenesis, vascular permeability, and inflammation [23, 24]. Aqueous ANGPTL4 was obviously increased in PDR, inducing retinal neovascularization [25, 26]. Lu et al. demonstrated that ANGPTL-4 regulated diabetic retinal inflammation and angiogenesis by, at least partly, activating profilin-1 both in human retinal microvascular endothelial cells and in diabetic rats. Moreover, the activation of the ANGPTL4 was dependent on the overexpression of its upstream regulatory factor, HIF-1*α* [27]. Using the oxygen-induced retinopathy mouse model for ischemic retinopathy, Xin et al. provided the evidence that hypoxic Müller cells promoted vascular permeability by HIF-1–dependent upregulation of ANGPTL4 [28]. They also observed that inhibition of ANGPTL4 expression reduced the angiogenic potential and vascular permeability of hypoxic retinal Müller cells, which was additive with inhibition of VEGF expression [26]. Further, they identified the ANGPTL4/NRP/RhoA pathway as a therapeutic target for DME [14]. In the present study, multiple regression analysis suggested that ANGPTL4 might be more sensitive for NPDR and DME than VEGF-A, as shown by increased RT, RV, and decreased DVD. Overall, we hypothesized that ANGPTL4 may participate in the pathogenesis of DME through the above pathways, and targeting both ANGPTL4 and VEGF may be essential for effective management of DME.

We evaluated macular perfusion using 3 × 3 mm scan mode of OCTA which divided retina into foveal and parafoveal area. Compared with DWR and NPDR/DME- group, the whole and parafoveal DVD in NPDR/DME+ group were significantly decreased. The foveal DVD decreased with the severities of the three groups, although there was no statistical significance. Consistently, AttaAllah et al. [29] observed a reduction of parafoveal DVD in NPDR eyes with DME compared with diabetic eyes without DME. Furthermore, Toto et al. investigated the changes of retinal vessel in DME compared with normal controls and found a decrease in foveal and parafoveal DVD, especially in parafoveal area [30]. Similarly, we observed an obvious change of DVD in parafoveal area but not in foveal area, which may attribute to different controls included. Besides, Simonett et al. found that parafoveal DVD was decreased in DWR and NPDR patients compared with nondiabetic controls, while no difference was found in SVD [31, 32], which was consistent with our study. All these evidences suggested that, except for DR, the parafoveal nonperfusion in DCP was also an early indicator for DME. However, a large amount of data will be needed to determine the normal reference range of DVD.

Our study had several limitations. First, PDR patients without DME were usually treated with laser in clinical practice, and their aqueous humor samples could not be collected. For this reason, PDR patients were not included in our study. Further studies including PDR patients with or without DME are needed to explore the role of ANGPTL4 in all types of DR. Second, the stages of DME were not grouped, and thereby, ANGPTL4 involved in early or late stage of DME could not be determined. Considering the small sample size in this study, large sample size and detailed classification of DME will be necessary. Third, our study only analyzed the OCTA images with 3 × 3 mm scan centered on the fovea, which could not reflect the blood flow of whole retina. Therefore, larger images should be analyzed in the future.

In conclusion, our study showed that microvascular change of NPDR patients with DME initially occurs at DCP with decreased vascular density in parafoveal area. The level of ANGPTL4 in aqueous humor was significantly increased in NPDR patients with DME, and ANGPTL4 was an influencing factor for RT, RV, and DCP, suggesting that ANGPTL4 may predict the progression of DME in NPDR patients.

## Figures and Tables

**Table 1:** Demographic characteristics of the 3 groups.

Characteristic	DWR (*N* = 16)	NPDR/DME- (*N* = 17)	NPDR/DME+ (*N* = 21)	*F*/*χ*^2^ value	*P* value (among the 3 groups)
Age (yrs)	67.94 ± 8.80	64.47 ± 7.19	64.76 ± 11.61	0.675	0.514^a^
Male gender, no. (%)	5 (31.25%)	7 (41.18%)	14 (66.67%)	5.046	0.080^b^
BMI	24.80 ± 3.16	24.19 ± 1.88	25.97 ± 3.27	1.895	0.161^a^
MAP	100.33 ± 7.41	105.02 ± 12.35	101.95 ± 11.07	0.844	0.436^a^
Fasting plasma glucose (mmol/l)	6.72 ± 1.43	7.42 ± 1.91	6.51 ± 1.32	1.667	0.199^a^
HbAlc (%)	7.48 ± 1.25	7.97 ± 1.48	7.56 ± 0.98	0.789	0.460^a^
Duration of diabetes (yrs)	7.88 ± 5.94	11.88 ± 4.96	11.20 ± 9.21	1.485	0.236^a^
NPDR classification				2.354	0.354^b^
Mild (%)	—	5 (29.41%)	4 (19.05%)		
Moderate (%)	—	8 (47.06%)	7 (33.33%)		
Severe (%)	—	4 (23.53%)	10 (47.62%)		

BMI: body mass index; MAP: mean arterial pressure; HbA1c: hemoglobin A1c; —: not analyzed. Values are mean ± standard deviation unless otherwise indicated. ^a^One-way analysis of variance with post hoc least significant difference multiple comparison tests. ^b^Chi-square test.

**Table 2:** Comparison of the cytokine levels in the aqueous humor among the 3 groups [M(Q1,Q3)].

Aqueous cytokines	DWR (pg/ml) (*N* = 16)	NPDR/DME- (pg/ml) (*N* = 17)	NPDR/DME+ (pg/ml) (*N* = 21)	*χ* ^2^ value	*P* value
HGF	445.64 (286.17, 583.95)	400.13 (353.31, 688.43)	626.10 (528.81, 805.84)^ab^	9.596	0.008
HB-EGF	1.18 (1.02, 1.62)	1.21 (0.87, 1.48)	1.06 (0.92, 1.27)	1.586	0.453
FGF-2	13.19 (11.17, 18.16)	15.75 (11.66, 19.81)	13.19 (9.33, 15.75)	1.803	0.406
FGF-19	50.29 (33.56, 66.44)	39.42 (32.11, 53.29)	234.78 (35.51, 335.39)^b^	8.362	0.015
Endothelin-1	8.15 (6.24, 13.59)	8.83 (5.97, 10.64)	8.22 (5.78, 10.23)	0.763	0.683
Leptin	68.33 (61.73, 80.97)	64.84 (57.66, 79.31)	88.26 (68.33, 135.53)	5.895	0.052
IL-8	4.64 (3.32, 7.42)	7.16 (3.67, 8.14)	9.97 (6.95, 17.18)^ab^	12.276	0.002
ANG-2	32.28 (26.52, 44.62)	36.52 (25.42, 44.92)	34.58 (25.13, 44.17)	0.014	0.993
ANGPTL4	1529.50 (1078.50, 4347.25)	2726.00 (2025.00, 12519.50)^a^	23778.00 (14490.00, 26311.50)^ab^	31.902	<0.001
PLGF	1.25 (1.02, 1.79)	1.23 (1.07, 3.54)	3.75 (2.74, 6.56)^ab^	20.631	<0.001
VEGF-A	179.14 (132.90, 220.41)	276.25 (159.75, 345.77)	363.93 (242.95, 564.12)^ab^	20.004	<0.001
VEGF-C	38.52 (29.76, 77.98)	45.36 (33.79, 65.16)	47.24 (34.26, 56.01)	0.076	0.963

HGF: hepatocyte growth factor; HB-EGF: heparin-binding EGF-like growth factor; FGF-2: fibroblast growth factor 2; FGF-19: fibroblast growth factor 19; IL-8: interleukin 8; ANG-2: angiopoietin-2; ANGPTL4: angiopoietin-like 4; PLGF: placental growth factor; VEGF-A: vascular endothelial growth factor-A; VEGF-C: vascular endothelial growth factor-C. Cytokine levels (pg/mL) are presented as median with interquartile range. The levels of cytokines in the aqueous humor of the 3 groups were compared by Kruskal-Wallis *H* test; one-to-one multiple comparisons were performed by Mann–Whitney *U* test, a: compared with control, ^a^*P* < 0.0167, b: compared with NPDR/ME- group, ^b^*P* < 0.0167.

**Table 3:** Comparison of the OCTA Metrics among the 3 groups (mean ± SD).

OCTA metrics	DWR (*N* = 16)	NPDR/DME- (*N* = 17)	NPDR/DME+ (*N* = 21)	*F* value	*P* value
RT (*μ*m)					
Fovea	240.90 ± 20.57	237.54 ± 22.77	331.76 ± 90.87^ab^	15.617	<0.001
Parafovea	308.59 ± 13.68	313.18 ± 20.56	379.25 ± 76.61^ab^	12.061	<0.001
RV (mm^3^)					
Fovea	0.19 ± 0.02	0.19 ± 0.02	0.26 ± 0.07^ab^	15.640	<0.001
Parafovea	1.90 ± 0.09	1.93 ± 0.14	2.26 ± 0.41^ab^	9.838	<0.001
SVD (%)					
Whole	39.37 ± 4.48	40.20 ± 3.63	39.31 ± 5.16	0.211	0.810
Fovea	12.24 ± 5.28	14.25 ± 5.24	15.04 ± 7.04	1.011	0.371
Parafovea	42.35 ± 4.86	42.56 ± 4.11	41.01 ± 5.80	0.535	0.589
DVD (%)					
Whole	49.05 ± 3.38	45.96 ± 4.54	42.35 ± 5.66^ab^	9.260	<0.001
Fovea	27.90 ± 5.24	25.08 ± 6.20	24.79 ± 9.06	0.962	0.389
Parafovea	52.25 ± 3.70	48.64 ± 5.19^a^	44.76 ± 5.94^ab^	9.752	<0.001
FD-300 vessel density (%)	43.57 ± 5.00	44.40 ± 5.19	42.54 ± 5.91	0.561	0.574
FAZ					
Area (mm^2^)	0.34 ± 0.08	0.37 ± 0.11	0.42 ± 0.25	1.074	0.349
Perimeter (mm)	2.37 ± 0.31	2.57 ± 0.42	2.82 ± 1.04	1.871	0.164
Acircularity index	1.16 ± 0.05	1.19 ± 0.06	1.23 ± 0.12	2.896	0.064

SD: standard deviation; RT: retinal thickness; RV: retinal volume; SVD: superficial vessel density; DVD: deep vessel densities; FAZ: foveal avascular zone. One-way ANOVA for normal distribution data among the 3 groups followed by post hoc least significant difference analysis between each two groups. a: compared with control; b: compared with NPDR/DME- group, *P* < 0.05 was deemed to be statistically significant.

**Table 4:** Correlation between cytokine levels in aqueous humor and OCTA metrics in NPDR eyes (*n* = 38).

	Foveal RT		Parafoveal RT		Foveal RV		Parafoveal RV		Whole DVD		Parafoveal DVD	
	*r*_*s*_	*P* value	*r*_*s*_	*P* value	*r*_*s*_	*P* value	*r*_*s*_	*P* value	*r*_*s*_	*P* value	*r*_*s*_	*P* value
HGF	0.250	0.130	0.211	0.203	0.249	0.132	0.127	0.447	-0.087	0.605	-0.144	0.389
FGF19	0.315	0.054	0.332	0.042	0.314	0.055	0.297	0.070	-0.256	0.121	-0.293	0.074
IL-8	0.175	0.293	0.158	0.345	0.172	0.302	0.041	0.807	-0.171	0.304	-0.184	0.268
ANGPTL4	0.569	<0.001	0.555	<0.001	0.566	<0.001	0.519	0.001	-0.352	0.030	-0.421	0.008
PLGF	0.363	0.025	0.352	0.030	0.365	0.024	0.326	0.046	-0.240	0.147	-0.288	0.080
VEGF-A	0.528	0.001	0.437	0.006	0.530	0.001	0.389	0.016	-0.373	0.021	-0.422	0.008

RT: retinal thickness; RV: retinal volume; DVD: deep vessel density. Spearman test, *P* < 0.05 was deemed to be statistically significant.

**(a):** 

Cytokines	Foveal RT				Parafoveal RT			
	*β*	SE	*P* value	*R* ^2^	*β*	SE	*P*value	*R* ^2^
ANGPTL4	4.299 × 10^−3^	0.745 × 10^−3^	<0.001	0.480	3.598 × 10^−3^	0.572 × 10^−3^	<0.001	0.523
VEGF-A	—	—	0.050		—	—	0.658	

**(b):** 

Cytokines	Foveal RV				Parafoveal RV			
	*β*	SE	*P* value	*R* ^2^	*β*	SE	*P* value	*R* ^2^
ANGPTL4	3.371 × 10^−6^	0.586 × 10^−6^	<0.001	0.479	17.705 × 10^−6^	3.285 × 10^−6^	<0.001	0.447
VEGF-A	—	—	0.050		—	—	0.602	

**(c):** 

Cytokines	Whole DVD				Parafoveal DVD			
	*β*	SE	*P* value	*R* ^2^	*β*	SE	*P* value	*R* ^2^
ANGPTL4	-1.705 × 10^−4^	0.611 × 10^−4^	0.008	0.178	-1.799 × 10^−4^	0.665 × 10^−4^	0.010	0.169
VEGF-A	—	—	0.148		—	—	0.238	

RT: Retinal thickness; RV: retinal volume; DVD: deep vessel density; —: without data from statistics software, multivariate linear regression models, *P* < 0.05 was deemed to be statistically significant.

## Data Availability

The data used to support the findings of this study are available from the corresponding author upon request.

## Supplementary Materials

Supplementary Materials Supplemental Table 1: single factor linear regression between cytokines and OCTA metrics. Single-factor linear regression showed that ANGPTL4 and VEGF-A were the influencing factors of RT, RV, and DVD (all, *P* < 0.05).
