# Supplementary material for: Aqueous Level of ANGPTL4 Correlates with the OCTA Metrics of Diabetic Macular Edema in NPDR
Source: J Diabetes Res. 2022 Jan 19;2022:8435603. doi: 10.1155/2022/8435603 (PMC8791715; doi:10.1155/2022/8435603)
Supplement: Supplementary Materials — Supplemental Table 1: single factor linear regression between cytokines and OCTA metrics. Single-factor linear regression showed that ANGPTL4 and VEGF-A were the influencing factors of RT, RV, and DVD (all, P < 0.05). [file 8435603.f1.docx]

| Supplemental Table 1: Single factor linear regression between cytokines and OCTA metrics (n=38). | | | | | | | | | |
| --- | --- | --- | --- | --- | --- | --- | --- | --- | --- |
| Cytokines | Foveal RT | | | |  | Parafoveal RT | | | |
|  | β | SE | *P* Value | *R*^2^ |  | β | SE | *P* Value | *R*^2^ |
| FGF19 | — | — | — | — |  | 0.032 | 0.034 | 0.357 | 0.024 |
| ANGPTL4 | 4.299×10^-3^ | 0.745×10^-3^ | <0.001 | 0.480 |  | 3.598×10^-3^ | 0.572×10^-3^ | <0.001 | 0.523 |
| PLGF | 2.913 | 3.33 | 0.388 | 0.021 |  | 1.697 | 2.683 | 0.531 | 0.011 |
| VEGF-A | 0.234 | 0.054 | <0.001 | 0.340 |  | 0.145 | 0.048 | 0.005 | 0.203 |
| Cytokines | Foveal RV | | | |  | Parafoveal RV | | | |
|  | β | SE | *P* Value | *R*^2^ |  | β | SE | *P* Value | *R*^2^ |
| ANGPTL4 | 3.371×10^-6^ | 0.586×10^-6^ | <0.001 | 0.479 |  | 17.705×10^-6^ | 3.285×10^-6^ | <0.001 | 0.447 |
| PLGF | 0.002 | 0.003 | 0.386 | 0.021 |  | 0.008 | 0.014 | 0.600 | 0.008 |
| VEGF-A | 0.184×10^-3^ | 0.043×10^-3^ | <0.001 | 0.340 |  | 0.738×10^-3^ | 0.258×10^-3^ | 0.007 | 0.186 |
| Cytokines | Whole DVD | | | |  | Parafovea DVD | | | |
|  | β | SE | *P* Value | *R*^2^ |  | β | SE | *P* Value | *R*^2^ |
| ANGPTL4 | －1.705×10^-4^ | 0.611×10^-4^ | 0.008 | 0.178 |  | －1.799×10^-4^ | 0.665×10^-4^ | 0.010 | 0.169 |
| VEGF-A | -0.011 | 0.004 | 0.009 | 0.176 |  | -0.011 | 0.004 | 0.018 | 0.145 |
| RT: Retinal thickness; RV: retinal volume; DVD: deep vessel density; —: not analyzed, multivariate linear regression models, *P*<0.05 was deemed to be statistically significant. | | | | | | | | | |
